# Supplementary material for: MetaRibo-Seq measures translation in microbiomes
Source: Nat Commun. 2020 Jun 29;11:3268. doi: 10.1038/s41467-020-17081-z (PMC7324362; doi:10.1038/s41467-020-17081-z)
Supplement: Supplementary file 10 — Supplementary Data 7 [file 41467_2020_17081_MOESM10_ESM.zip › File2/Confidence_High_Taxonomy/141266_out.krona.html]

Javascript must be enabled to view this page.

members
magnitude
magnitudeUnassigned
count
unassigned
taxon
rank

141266\_out

10

superkingdom
2
10

phylum
32066
10

203490
10
class

10
203491
order

family
10
203492

10
848
genus
1

SRS062540\_contig\_number\_contig-100\_5292.5292

6
851
species
2

SRS018479\_contig\_number\_contig-100\_21273.154064.154064SRS057619\_contig\_number\_contig-100\_21003.21004

155615
1
subspecies

SRS044366\_contig\_number\_169

subspecies
76859
1

SRS045015\_contig\_number\_contig-100\_8211.73124


SRS015755\_contig\_number\_20SRS104291\_contig\_number\_2194
76857
2
subspecies

species
860
3

SRS024318\_contig\_number\_12914SRS143039\_contig\_number\_contig-100\_1253.1254SRS147887\_contig\_number\_contig-100\_19168.19168
